# Huaier shows anti‐cancer activities by inhibition of cell growth, migration and energy metabolism in lung cancer through PI3K/AKT/HIF‐1α pathway

**DOI:** 10.1111/jcmm.16215

**Published:** 2020-12-30

**Authors:** Xiangli Liu, Lidan Liu, Keyan Chen, Lei Sun, Wenya Li, Shuguang Zhang

**Affiliations:** ^1^ Department of Thoracic Surgery the First Affiliated Hospital of China Medical University Shenyang China; ^2^ Department of Anesthesiology Shengjing Hospital of China Medical University Shenyang China; ^3^ Department of Laboratory Animal Science China Medical University Shenyang China

**Keywords:** AKT, HIF‐1α pathway, Huaier, NSCLC, energy metabolism, PI3K

## Abstract

Huaier has been verified to have anti‐cancer effects on many tumours. However, little information is available about the effects of Huaier on non‐small cell lung cancer (NSCLC). We sought to probe the anti‐cancer effects and related mechanisms of Huaier on lung cancer. A549 cells were pre‐treated with 2, 4 and 8 mg/mL Huaier at different time points. Thereafter, cell viability was analysed by CCK‐8 and the migration and invasion were detected by Scratch test and Transwell chamber migration assay. Moreover, ELISA, Western blot, shRNA transfection and RT‐PCR were conducted to discover the related gene and protein expressions of energy metabolism and phosphatidylinositol 3‐kinase (PI3K)/AKT/hypoxia‐inducible factor 1α (HIF‐1α) pathway. Furthermore, tumour xenografts were accomplished to inspect the anti‐cancer effects of Huaier. Our consequences suggested that Huaier considerably repressed cell viability and migration in a dose‐dependent way. In addition, Huaier statistically suppressed glycolysis, glucose transport and lactic acid (LA) accumulation. Besides, we detected that Huaier could inactivate the PI3K/AKT/HIF‐1α pathway. The in vivo data confirmed that Huaier obviously decreased tumour volume and tumour growth, reduced the glycolysis, glucose transport and HIF‐1α expression in the tumour‐bearing tissues. Our results suggested Huaier revealed anti‐tumour effects in both in vivo and in vitro possibly through PI3K/AKT/HIF‐1α pathway.

**Highlights**

1. Huaier significantly represses cell viability and migration in a dose‐dependent way
2. Huaier statistically suppresses glycolysis, glucose transport and LA accumulation
3. Huaier inactivates the PI3K/AKT/HIF‐1α pathway
4. Huaier obviously decreases tumour volume and tumour growth
5. Huaier reveals anti‐tumour effects possibly through PI3K/AKT/HIF‐1α pathway.

## 1 INTRODUCTION

Lung cancer is a well‐known heterogeneous disorder, involving at least 50 histomorphological subtypes.^1^, ^2^, ^3^ Non‐small cell lung cancer (NSCLC) was recognized as the largest proportion of all lung cancer cases over the past years, accounting for approximately 80%‐85%.^4^ Clinically, at least 60% of lung cancer individuals are diagnosed at the middle and locally advanced phases, when the malignancy cannot be treated by surgical resection.^5^ Currently, conservative treatments including chemotherapy and radiation have been the foremost ways for individuals with lung cancer.^5^ However, emerging evidence shows that novel immunotherapy might be more effective in some patients; the side‐effects cannot be ignored.^6^, ^7^

Traditional Chinese medicine (TCM) was largely applied for malignancy controlling for a long time in China, including NSCLC.^8^, ^9^ TCM is extremely popular because of its rich in natural resources, high efficiency, slight toxicity and varied chemical components. Trametes robiniophila Murr (Huaier) is acknowledged as a sandy beige mushroom and also an official fungi originated on the trucks of trees and was put into use as one key TCM for approximately 1600 years.^10^ The foremost active component of Huaier is a polysaccharide glycoprotein. It is a mixture of six types of monosaccharides, consisting of polysaccharides, amino acids and water. The anti‐tumour beneficial achievement of Huaier has been used as a complementary therapy in diverse cancers, such as colon cancer, ovarian cancer, prostate cancer, gastric cancer, breast cancer and hepatocellular carcinoma.^10^, ^11^, ^12^, ^13^, ^14^, ^15^, ^16^ Nonetheless, the effects of Huaier on NSCLC have been endured unwell unstated. Metabolism is one basic feature of the body's life activities, including substance metabolism and the accompanying energy metabolism.^17^ Energy metabolism is a process that an organism releases, converses and utilizes energy during substance metabolism. The energy metabolism is different in tumour cells from that of normal cells.^18^ Even under sufficient oxygen supply, tumour cells still acquire energy mainly through glycolysis, known as aerobic glycolysis, producing a large amount of lactic acid (LA) and a small amount of ATP, which is termed as ‘Warburg effect’.^19^ In recent years, the molecular mechanism of tumour pathogenesis has been increasingly understood, and more and more attention has been paid to the energy metabolism of tumour cells.^20^, ^21^, ^22^ Abnormal energy metabolism has become one of the markers of an increasing number of tumour cells.^23^ However, little information is available about use of TCM to regulate the energy metabolism of tumour cells.

Phosphatidylinositol 3‐kinase (PI3K)/AKT signalling pathway has been demonstrated to participate in diverse cellular activities and pathological mechanisms. For example, activation of the PI3K/AKT pathway plays a critical role in meditating many tumour angiogenesis, invasion and metastasis, including lung cancer.^24^, ^25^ Hypoxia‐inducible factor 1α (HIF‐1α) is a nuclear transcription factor that is specifically active under hypoxia and s regulated by oxygen concentration.^26^, ^27^ It has also been considered as the initiator factor of the endogenous protective mechanism and is a downstream factor of the PI3K/AKT signalling pathway.^28^, ^29^ Nonetheless, it is still unclear whether the effects of Huaier on NSCLC cells are implicated in PI3K/AKT/HIF‐1α signalling pathway.

In the current study, we inspected the functional efficiency of Huaier on NSCLC cell line A549 and a nude mouse xenograft model, as well as its potential mechanism about the energy metabolism. Our data demonstrated that Huaier exerted an anti‐tumour impact both in vivo and *in vitr*o. Our study might offer a novel therapy for lung cancer.

## 2 MATERIALS AND METHODS

### 2.1 Cell culture and drug preparation

A549 cells were obtained from American type culture collection (ATCC, Manassas). These cells were incubated in high‐glucose Dulbecco's modified Eagle's medium (DMEM, Gibco) containing 10% foetal bovine serum (FBS, Gibco) in a 5% CO_2_ incubator at 37°C. Huaier (10 g, Sigma‐Aldrich) was completely lysed in 100 mL medium and filtered with a 0.25 μm filter to prepare as 100 g/mL of stock solution. Samples were then diluted into 2, 4 and 8 mg/mL working fluid before use. Cells in the control group were incubated with an equal volume of high‐glucose complete DMEM. In addition, an activator EGF (10 ng/mL, Sigma‐Aldrich) and an inhibitor Wortmannin (2 μg/mL, Sigma‐Aldrich) of PI3K/AKT signalling pathway were performed to the A549 cells.

### 2.2 Cell Counting Kit‐8 (CCK‐8)

The cells in the logarithmic stage were digested with trypsin and diluted into 1‐5 × 10^4^ cells/ml. Cells (1‐5 × 10^3^ cells/well) were plated onto the 96‐wells and incubated at 37°C overnight. CCK‐8 and serum‐free DMEM were mixed at volume ratio of 1:10. Then, the mixture (100 µL/well) was administrated to the wells and incubated at 37°C for 1 hour in 5% CO_2_ incubator. Absorbance (*A*) value was assessed at 450 nm using a microplate spectrophotometer. The proliferation inhibition rate (%) was equal to 1 − *A* values in the treatment group/*A* values in the non‐treatment group × 100%.

### 2.3 Scratch test

Scratch test was accomplished to analyse the migration ability of the treated cells. After treatment, the cells were digested and 8 × 10^5^ cells were incubated in 35 mm^2^ Petri dish. A mark line was made in the bottom of the disc with a marker pen. After absorbing the culture medium, cells in the disc were marked off with a 200 μL pipet tip perpendicular to above‐mentioned marker. The removed cells were rinsed with phosphate‐buffered saline (PBS). Afterwards, cells were further nurtured with serum‐free medium and photographed at 48 hours. The intersection of the line marked by the marker pen and the cell scratch was considered as an observation point for fixed‐point observation.

### 2.4 Transwell chamber migration assay

Artificial basement membrane was made with Matrigel glue (Millipore). After treatment with Huaier for 24 hours, A549 cells were rinsed twice with PBS and resuspended with the complete medium. After adjusting of the cell density, 1 × 10^5^ cells were added in the upper chamber, with a total volume of 0.5 mL. High‐glucose DMEM comprising 10% FBS was put in the lower chamber. Crystal violet staining was performed after 48 hours of incubation in the 37°C incubator. Three fields were randomly selected under a 200 × light microscope to quantify the number of cells crossing artificial basement membrane, and the average value was calculated.

### 2.5 Glucose consumption test

The A549 cells were seeded in a 6‐well plate with culture medium in the absence of phenol red‐5 mmol/L glucose. At different time points of culture, 50 μL culture medium was aspirated from conditioned medium and diluted with distilled water. The concentration of glucose was measured using the glucose assay kit (Sigma‐Aldrich) based on the manufacturer's directions. The samples were then mixed with the glucose assay substance and incubated at 37°C for half an hour. Optical density at 540 nm was measured. The amount of glucose consumed during inoculation was determined by deducting the quantity of glucose remaining in the fresh cell culture medium at the designated time points from the initial amount of glucose.

### 2.6 Enzyme‐linked immuno sorbent assay (ELISA)

ELISA was performed to assess the amount of hexokinase II (HK‐II), phosphofructokinase (PFK), pyruvate kinase (PK) and glucose transporter 1 (GLUT1) in the animals according to the kit instructions (USCN). Standard dilutions (50 μL) were added to the untreated cell well, while 50 μL of detection reagent A was added to each well immediately. After incubation for 1 hour at 37°C, the solution was aspirated and washed with 350 μL of 1 × Wash Solution to each well for 1‐2 minutes. Thereafter, the residual liquid was totally detached by cracking the plate onto absorbent paper. Subsequently, detection reagent B working solution (100 μL) was performed to each well and nurtured for half an hour at 37°C, followed by adding of 90 μL of substrate solution to each well. After incubation for 10‐20 minutes at 37°C, stop solution (50 μL) was administrated to each well. Finally, the samples were run on a microplate reader at 450 nm immediately.

### 2.7 Western blot

Entire protein was taken out from the cells by RIPA buffer (Sigma‐Aldrich). After harvesting and lysis, the protein density was calculated using a BCA kit (Beyotime Institute of Biotechnology) according to protein standard curves. Samples (20 μg) were loaded in each well, electrophoresed and transferred to the PVDF membrane (Thermo Scientific). The membrane was then congested with 5% bull serum albumin (BSA, Thermo Scientific) at room temperature for 1 hour, washed, nurtured with the following primary antibodies at 4°C overnight: anti‐HK‐II (ab104836, Abcam), anti‐PFK (ab37583, Abcam), anti‐PK (ab85555, Abcam), anti‐AKT (ab179463, Abcam), anti‐p‐AKT (ab81283, Abcam), anti‐HIF‐1α (ab51608, Abcam) and anti‐GLUT1 (ab115730, Abcam). GAPDH was performed as an internal control (ab9485, Abcam). Thereafter, the membranes were washed and nurtured with secondary antibody at room temperature for 1 hour. Positive bands were exposed and visualized by BeyoECL Plus (Beyotime) and quantified by Image Lab™ software (Bio‐Rad).

### 2.8 Transfection

The HIF‐1α shRNA plasmid was acquired from GenePharma (Shanghai, China). A549 cells (1.5 × 10^5^ cells/mL) were seeded and then transfected with the HIF‐1α shRNA plasmid using Lipofectamine 2000 (Invitrogen) following to the constructor's references. The cells stably expressing shRNA were maintained in the occurrence of 0.5‐1.0 μg/mL puromycin. The culture media was harvested and got through a 0.45 μm litre when the cells extended approximately 70%‐80% confluence. The virus stock and polybrene were then put straight to the target cells.

### 2.9 RT‐PCR

The A549 cells were homogenized by addition of 1 mL of Trizol reagent (Invitrogen) to extract total RNA. OD260 values were measured, and RNA concentration was calculated. All samples were stored at −80°C. Real‐time quantitative PCR was conducted with the following methods: pre‐degeneration at 95°C for 10 seconds, degeneration at 95°C for 15 seconds, annealing at 56°C for 20 seconds and extension at 72°C for 30 seconds, totally 45 cycles, followed by extension at 72°C for 10 minutes, termination reaction at 4°C. β‐actin was used as an internal control.

### 2.10 Establishment of a nude mouse xenograft model

Xenograft model was constructed by using the 4‐5 weeks old male BALB/c athymic nude mice (n = 12; 16‐18 g) (Shanghai Slac Laboratory Animals Ltd.) and was maintained at specific pathogen‐free (SPF) conditions in the Laboratory Animal Center of China Medical University (Shenyang, China). The animal experiment was permitted by the Ethics Committee of the First Affiliated Hospital of China Medical University. For the treatment, the A549 human lung cancer cells were inoculated subcutaneously into the armpit of nude mice. Twenty‐four hours after inoculation, the nude mice were randomly distributed into an experimental group and a control group, with six mice in each group. Mice in the experimental group were administered Huaier granule (2.5 g/kg, ig) and those in the control group were given equal amount of normal saline for 40 successive days. The tumour volume and weight were calculated to evaluate the effects of Huaier on the cancer growth.

### 2.11 Immunohistochemistry (IHC)

The HIF‐1α in the tumour samples was measured by IHC. In brief, all the specimens were fixed with 40 g/L buffered formaldehyde, paraffin‐embedded and then continuously sliced into 5‐µm sections. Then, the sections were attached to poly‐L‐lysine coated glass slides and incubated overnight at 60°C. Thereafter, sections were dewaxed, rehydrated, treated with antigen retrieval solution at room temperature for 10 minutes and washed with 0.1 mol/L PBS for three times. Then, the sections were treated with normal goat serum blocking solution (Beyotime) at room temperature for 20 minutes and with primary antibody overnight at 4°C. Afterwards, the slides were washed and treated with biotinylated secondary antibody (IgG) (Vector Laboratories) for 20 minutes at 37°C, washed and treated with horseradish enzyme‐conjugated streptavidin (Thermo Fisher Scientific) for 20 minutes at 37°C. Immunoreacted cells were visualized with 3, 3'‐diaminobenzidine (DAB, Sigma), counterstained with haematoxylin, mounted with neutral gum and finally observed under a microscope.

### 2.12 Statistical analysis

Measurement data are presented as the mean ± SD. The statistics was examined using *t* test for two groups or a one‐way analysis of variance (ANOVA) followed by post hoc test for three or more groups with SPSS 19.0 software. A value of *P* < .05 was regarded as statistically significant.

## 3 RESULTS

### 3.1 Huaier suppresses lung cancer A549 cell viability and migration

**FIGURE 1:**
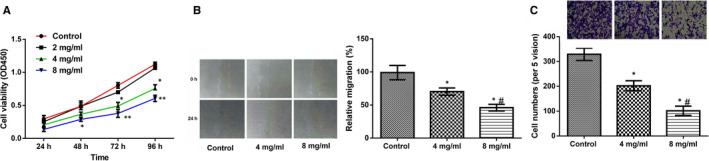
Huaier suppress lung cancer A549 cell viability, migration and invasion. Huaier (2, 4 and 8 mg/mL) was used to treat lung cancer A549 cells for 24, 48, 72 and 96 h. (A) Huaier obviously decreased on A549 cell viability in a dose‐dependent manner; (B‐C) Huaier evidently decreased the migration ability of A549 cells in a dose‐dependent manner. *Compared with treat for 24 h, *P* < .05; #compared with treat for 48 h, *P* < .05

To reveal the functional effects of Huaier on lung cancer, we performed different doses of Huaier (2, 4 and 8 mg/mL) to lung cancer A549 cells for 24, 48, 72 and 96 hours. CCK‐8 was used to analyse the effect of Huaier on A549 cells viability. Results displayed that cell viability was gradually and significantly decreased in the 4 and 8 mg/mL Huaier groups at 72 hours and 96 hours in A549 cells (*P* < .05 or *P* < .01). However, the cell viability was no obvious in the 2 mg/mL group (Figure 1A). Therefore, we chose 4 and 8 mg/mL as the experimental group in the next experiment. Further we analysed the effects of Huaier on the migration ability using scratch test and Transwell chamber migration assay. As shown in Figure 1B,C, the results confirmed that compared to the control group, the healing ability of cells was significantly enhanced in a dose‐dependent manner (*P* < .05), while the number of migration cells were statistically diminished in a dose‐dependent way (*P* < .05).

### 3.2 Huaier inhibits A549 cells glycolysis, glucose transport and LA accumulation

**FIGURE 2:**
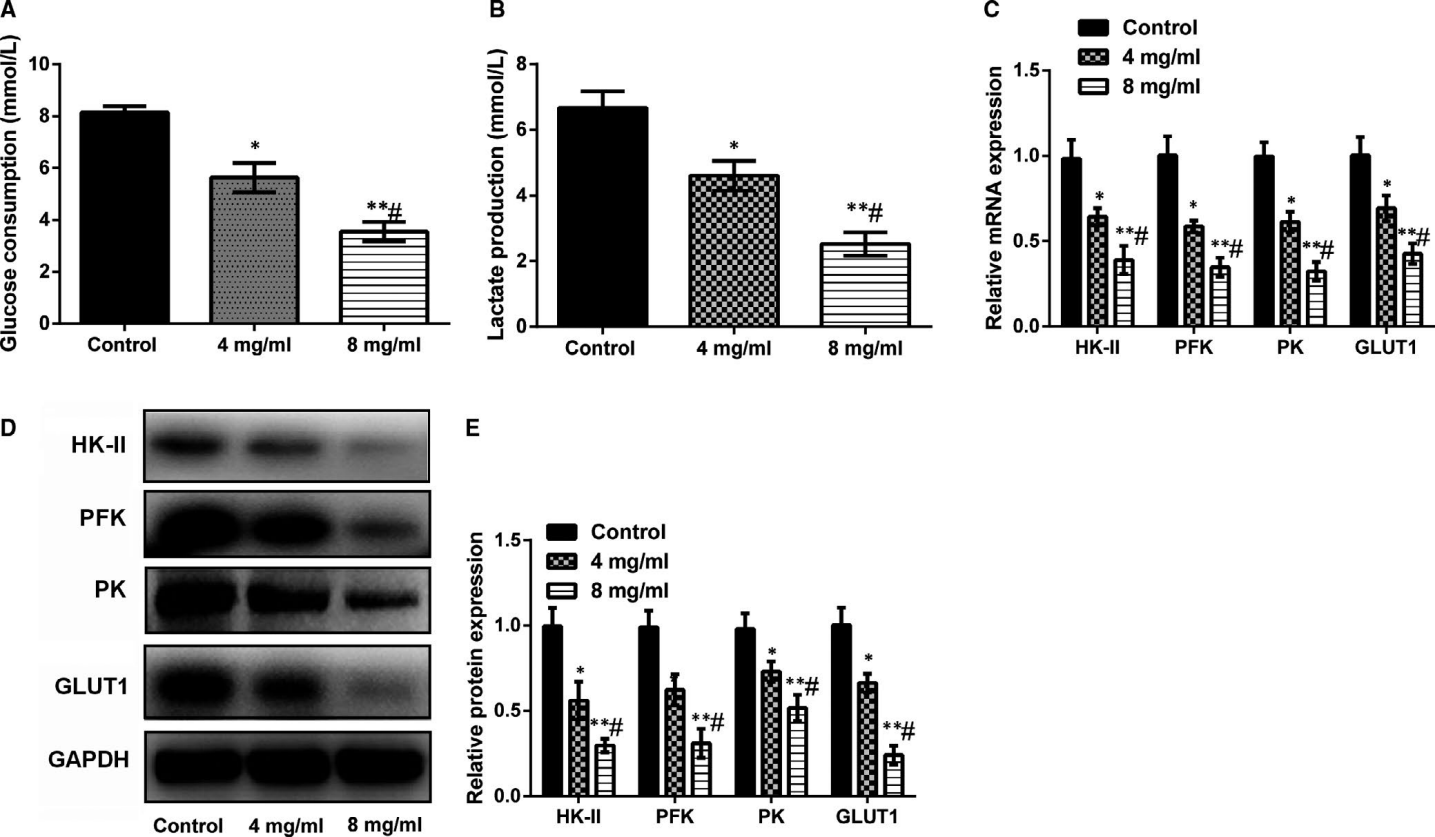
Huaier inhibits A549 glucose metabolism. Huaier (2, 4 and 8 mg/mL) was used to treat lung cancer A549 cells for 24 h. (A), Huaier markedly decreased the glucose consumption in a dose‐dependent manner; (B) Huaier distinctly diminished the LA accumulation; (C‐E) Huaier conspicuously lessened the key enzymes for glucose metabolism in both mRNA and protein levels. HK‐II, Hexokinase II; PFK, phosphofructokinase; PK, pyruvate kinase; GLUT1, glucose transporter 1; LA, lactic acid. Compared with control group, *P < .05 or **P < .01; #compared with 4 mg/mL group, *P* < .05

It has been well‐acknowledged that cell viability is closely related to its energy status. Malignant tumours often grow rapidly with increased intake of glucose, increased glycolytic activity and accumulation of LA. Therefore, we explored the effects of Huaier on A549 cells metabolic capability. As demonstrated in Figure 2A,B, our data verified that Huaier (4 and 8 mg/mL) could significantly decrease the glucose uptake and LA accumulation in a dose‐dependent way compared with the control group, the glucose uptake and lactate production were considerably lessened (*P* < .05 or *P* < .01). Next, we investigated the underlying mechanism of the glycometabolism by measuring the key enzymes of glycolysis (HK‐II, PFK and PK) and GLUT1 that helps the transport of glucose across the plasma membranes of mammalian cells. Western blot and RT‐PCR results suggested that, compared with the control group, all the protein and mRNA expression levels of HK‐II, PFK, PK and GLUT1 were significantly decreased in the Huaier group in a dose‐dependent way (*P* < .05 or *P* < .01, Figure 2C‐2E). The above results indicated that Huaier inhibited the glycolysis and glucose transport in A549 cells.

### 3.3 Huaier inactivates PI3K/AKT/HIF‐1α pathway

**FIGURE 3:**
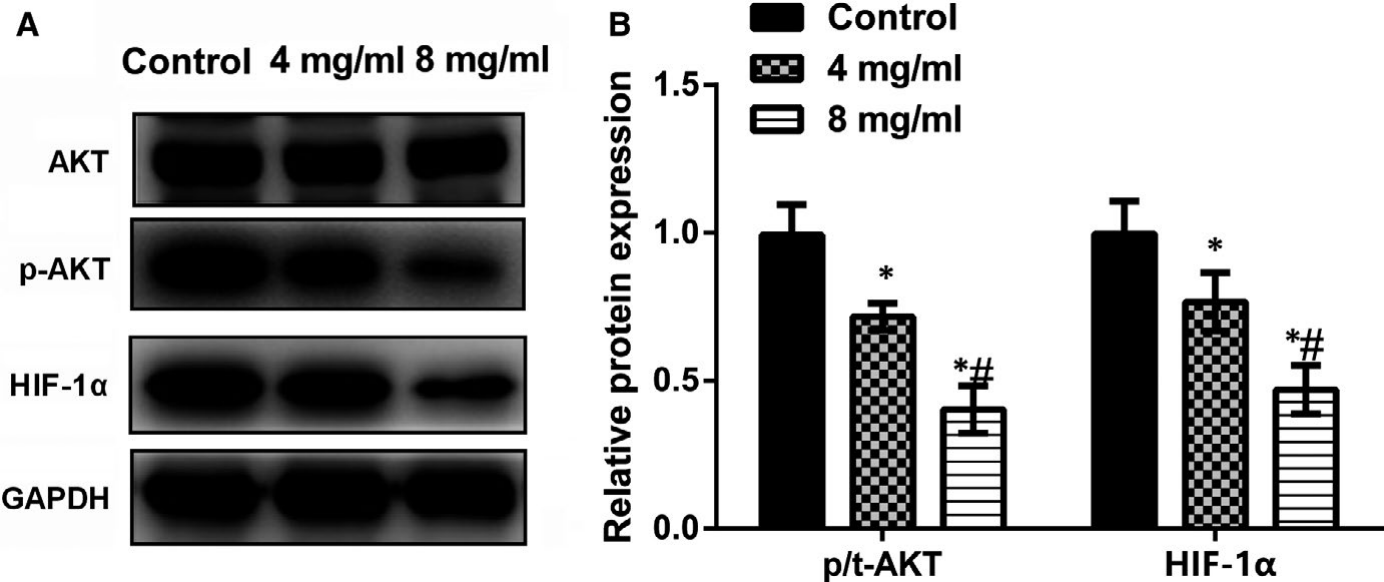
Huaier regulates PI3K/AKT/HIF‐1α pathway. Huaier (4 and 8 mg/mL) was used to treat lung cancer A549 cells. (A‐B) Huaier obviously decreased the protein expression of p‐AKT and HIF‐1α. PI3K: phosphatidylinositol 3‐kinase; HIF‐1α, hypoxia‐inducible factor‐1. *Compared with control group, *P* < .05; #Compared with 4 mg/mL group, *P* < .05

Much evidence shows that PI3K/AKT pathway plays an imperative part in energy metabolism and tumour growth. Thus, we also detected whether Huaier reveals its anti‐tumour functions by regulating this pathway. Western blot results displayed that the protein expression of p‐AKT and HIF‐1α was significantly declined in a dose‐dependent way in the Huaier group (*P* < .05, Figure 3A,B) compared with the control group. These results proposed that Huaier effectively inactivated PI3K/AKT/HIF‐1α signalling pathway.

### 3.4 Huaier reverses the energy metabolism via inhibiting PI3K/AKT/HIF‐1α pathway in A549 cells

**FIGURE 4:**
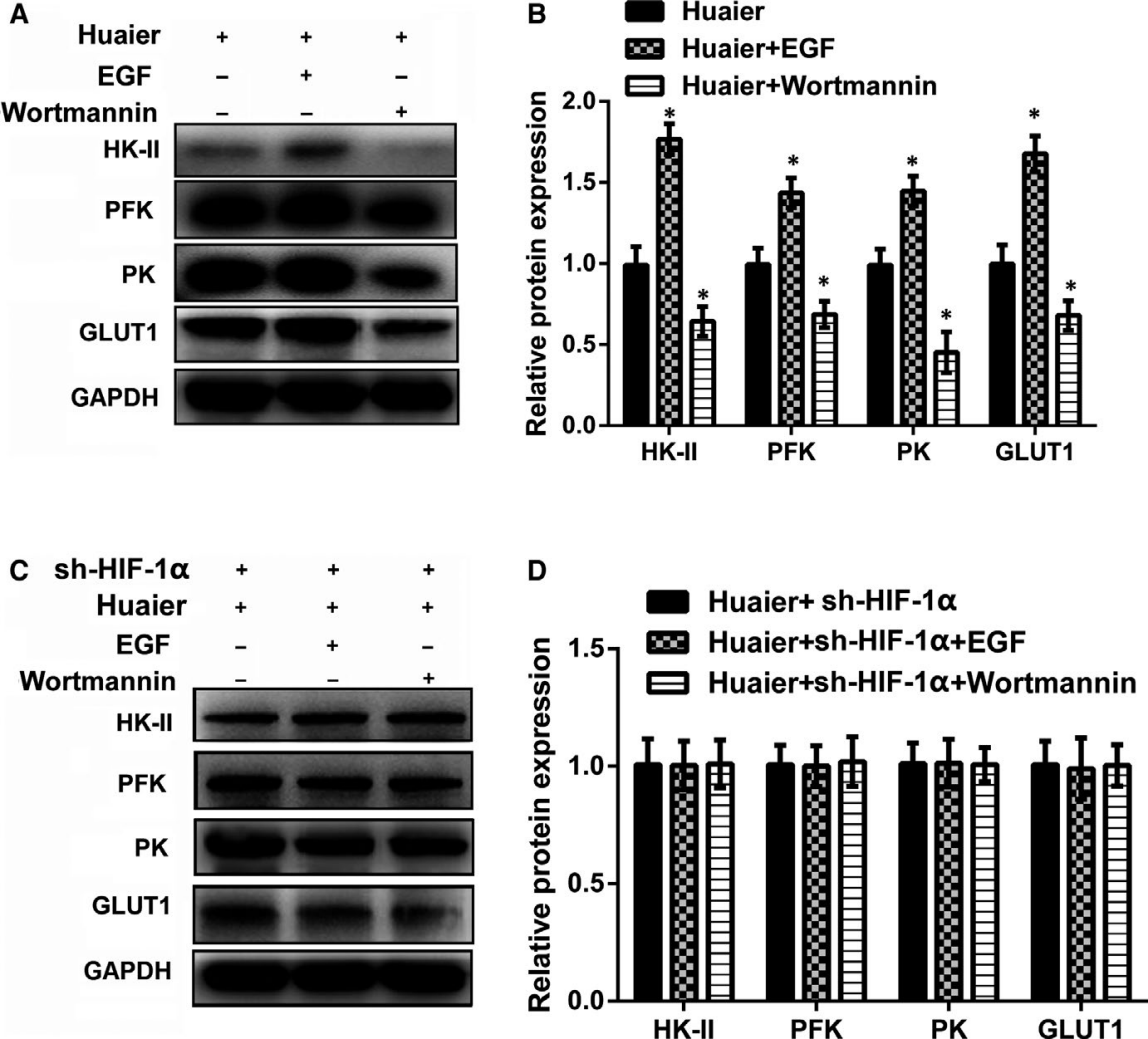
Huaier reverses the energy metabolism in A549 via inhibiting PI3K/ AKT/HIF‐1α signalling pathway. Huaier (4 mg/mL), EGF (10 ng/mL) and Wortmannin (2 μg/mL) were used to investigate the possible mechanism of Huaier reversal on energy metabolism. (A‐B) EGF significantly increased the protein expression of key enzymes for glucose metabolism, while Wortmannin showed the opposite results; (C‐D) silencing of HIF‐1α reversed the energy metabolism in A549 cells. HK‐II, Hexokinase II; PFK, phosphofructokinase; PK, pyruvate kinase; GLUT1, glucose transporter 1; PI3K: phosphatidylinositol 3‐kinase; HIF‐1α, hypoxia‐inducible factor‐1; EGF, epidermal growth factor. *Compared with Huaier group, *P* < .05

Subsequently, we explored whether the effects of Huaier on the energy metabolism via inhibiting PI3K/AKT/HIF‐1α pathway by administration of the activator (EGF) and inhibitor (Wortmannin) of the pathway. Western blot data suggested that treatment with EGF significantly increased the protein expression of HK‐II, PFK, PK and GLUT1 (*P* < .05), and treatment with Wortmannin significantly decreased the protein expression of HK‐II, PFK, PK and GLUT1 (*P* < .05, Figure 4A, B). However, the protein expression of HK‐II, PFK, PK and GLUT1 was not obvious after transfection with sh‐HIF‐1α (Figure 4C, D). These results implied that Huaier reversed the energy metabolism via inhibiting PI3K/AKT/HIF‐1α pathway in A549 cells.

### 3.5 Huaier inhibits the growth of tumour xenografts in nude mice

**FIGURE 5:**
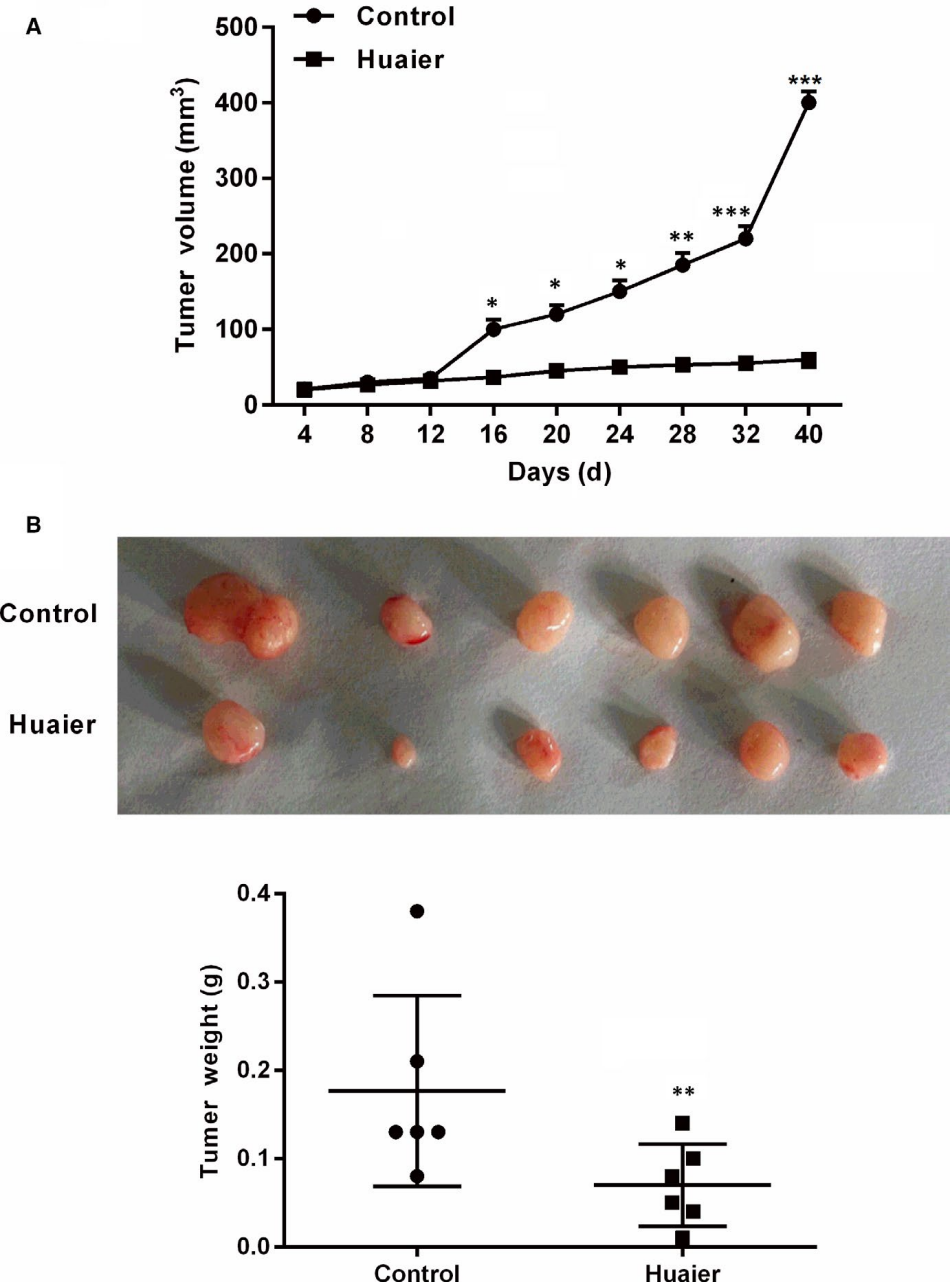
Huaier inhibits the growth of tumour xenografts in nude mice. A549 cells were inoculated subcutaneously into the armpit of nude mice. The mice in the Huaier group received Huaier (2.5 g/kg, ig) and those in the control group were given equal amount of normal saline for 40 successive days. (A) Huaier markedly decreased the tumour growth volume. (B) Huaier distinctly diminished the weight of tumour. Compared with control group, *P < .05, **P < .01 or ***P < .001

To further investigate the role of Huaier in living tissue, tumour xenograft was performed in the nude mice. Forty days after administration with Huaier, we detected the tumour volume and weight. As displayed in the Figure 5A, we observed that the tumour volume was considerably lower in the Huaier group than that in the control group (*P* < .05, *P* < .01 or *P* < .001). At the end of the experiment, the total weight of tumour xenografts in the Huaier group was suggestively smaller than that in the control group (*P* < .01, Figure 5B). These findings confirmed that Huaier greatly inhibited the growth of tumour xenografts.

### 3.6 Huaier inhibits the energy metabolism of tumour xenografts in nude mice

**FIGURE 6:**
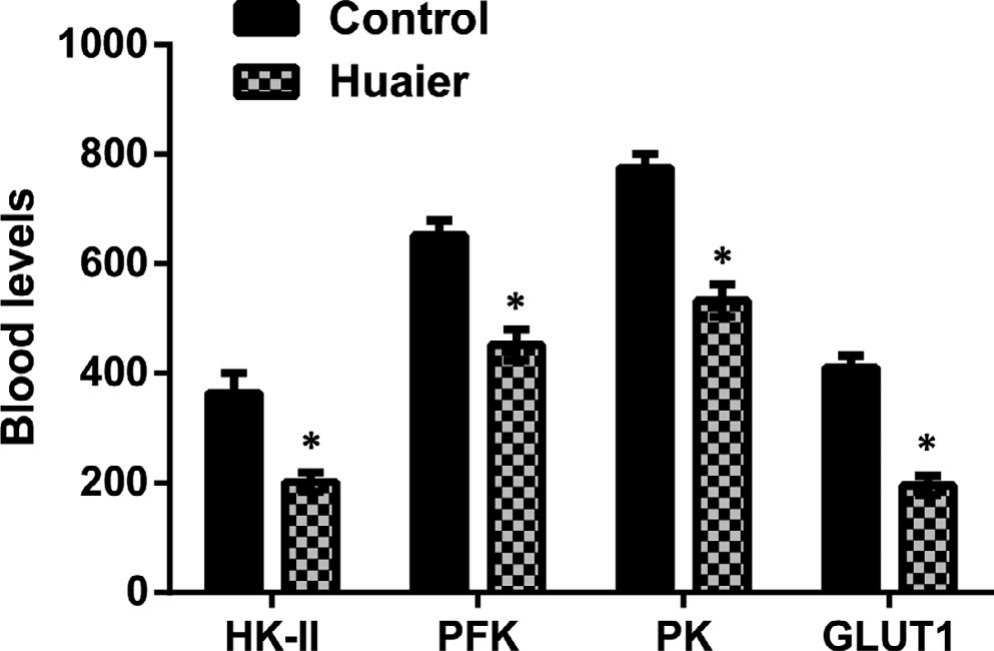
Huaier inhibits the energy metabolism of tumour xenografts in nude mice. ELISA was used to analyse HK‐II, PFK, PK and GLUT1 levels. The results showed that Huaier could ominously decrease the protein expression of HK‐II, PFK, PK and GLUT1 levels. HK‐II, Hexokinase II; PFK, phosphofructokinase; PK, pyruvate kinase; GLUT1, glucose transporter 1; ELISA, enzyme‐linked immunosorbent assay. *Compare with control group, *P* < .05

We further explored the effects of Huaier on HK‐II, PFK, PK and GLUT1 levels in the tumour‐bearing tissue. As exposed in Figure 6, our consequences suggested that the levels of HK‐II, PFK, PK and GLUT1 were ominously lessened in the Huaier group compared with the control group (*P* < .05). Our in vivo data were in line with the in vitro data, indicating that Huaier could inhibit the glycolysis in the lung tumour.

### 3.7 Huaier decreases the expression of HIF‐1α in the tumour‐bearing tissues

**FIGURE 7:**
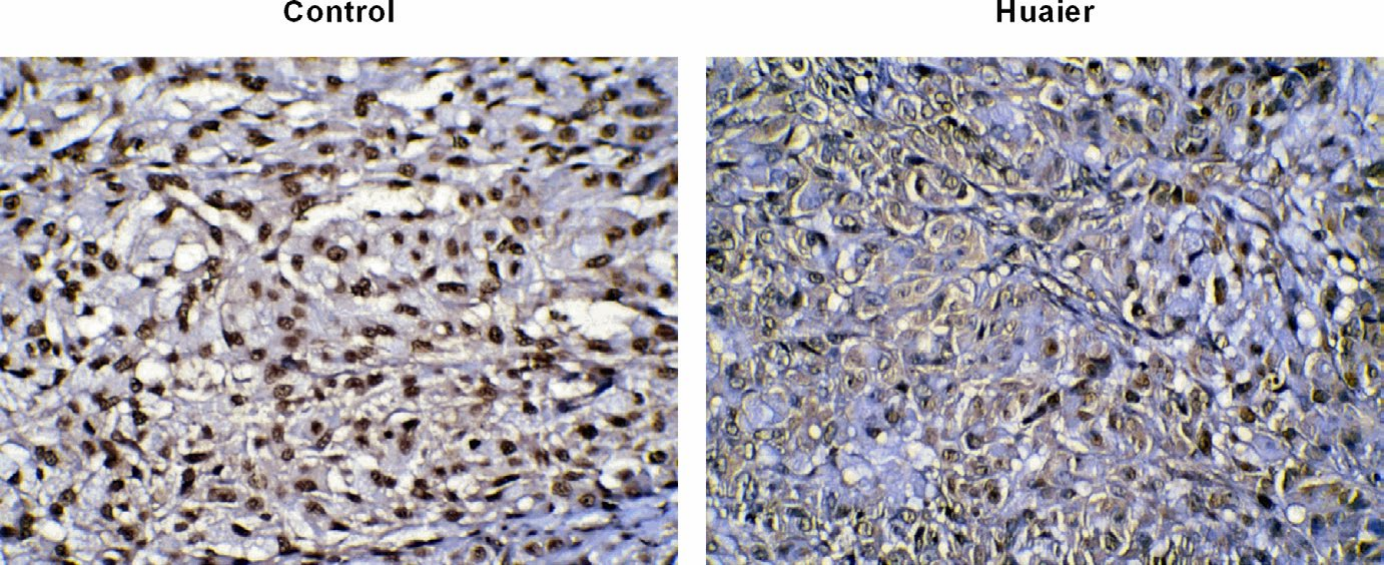
Huaier diminishes the expression of HIF‐1α in tumour‐bearing tissues. IHC was used to analyse HIF‐1α expression in both the control and Huaier group. The expression of HIF‐1α was significantly lessened. HIF‐1α, hypoxia‐inducible factor‐1; IHC, immunohistochemistry

The results of IHC presented that the expression of HIF‐1α was significantly obvious in the control group, with the strong nuclear staining (dark tan) and also stained in cytoplasm. Nonetheless, the expression of HIF‐1α in the Huaier group was significantly reduced, with the decrease of nuclear staining and cytoplasm staining (Figure 7).

## 4 DISCUSSION

In the current study, we confirmed that Huaier could constrain the proliferation, invasion and metastasis of lung adenocarcinoma A549 cells. In addition, our data suggested that Huaier could prevent the glucose metabolism of A549 cells, showing a decrease in glucose uptake and in lactate content. Moreover, we observed that Huaier markedly inactivated the PI3K/AKT/HIF‐1α pathway. For the in vivo experiments, the data suggested that administration of Huaier obviously inhibits the tumour volume and weight, as well as the decrease of glucose metabolism level and HIF‐1α staining in tumour‐bearing tissues. Our findings provided a novel innovative function of Huaier on lung cancer.

Recent research has established that Huaier can impede the growth of many tumours through various ways.^16^, ^30^, ^31^ An early study found that Huaier may constrain the proliferation and metastasis of lung cancer cells.^32^ Interestingly, we also found that Huaier exerted anti‐proliferation and inhibition of migration in A549 cells in a dose‐dependent way. However, little information is available about the impacts of Huaier on energy metabolism of lung cancer cells. Therefore, we aimed to explore this mechanism.

Anaerobic glycolysis has been well known to participate in the development and progress of malignancy. Hypoxia is the main cause of glycolysis in normal cells ^33^ and a normal condition in solid tumours.^34^ When the oxygen supply of tumour cells is insufficient, even without DNA mutation, mitochondrial respiratory chain dysfunction can be promoted. It utilizes glycolysis to produce ATP while inhibiting aerobic respiration.^34^ To some extent, the enhancement of glycolysis can be considered as the adaptability of tumour cells to hypoxic conditions. Therefore, we explored whether Huaier impeded the anaerobic glycolysis. Several glycolytic enzymes have been frequently augmented in malignancy cells. For instance, HK, an important enzyme that transforms glucose to glucose 6‐phosphate (G6P), is associated with transcription regulation, whose level is frequently increased in tumour cells.^35^ HK‐II has been confirmed to be involved in the raised glycolysis.^36^ PFK is a degree‐controlling enzyme that catalyses the step of glycolysis, which was recognized to be elevated in types of lung cancer.^37^ PK is another key regulator of glycolysis, playing critical roles in malignancy metabolism and tumour growth.^38^ In addition to the above enzymes, GLUT1 is a membrane protein carrier that facilitates the transport of glucose molecules from high concentration to low concentration across the membranes of the cells.^39^ Abnormally increased GLUT1 promotes glucose uptake, which is one of mechanisms by which tumour cells intake high level of glucose, showing key role in strengthening the glycolysis of tumour cells.^40^ GLUT1 has been reported to be highly expressed in lung cancer, and its expression is closely related to the poor prognosis of lung cancer.^41^ The enhancement of anaerobic glycolysis causes the LA accumulation, promoting the progress of cancer.^42^ Surprisingly, we found that Huaier could statistically diminish the glucose consumption, LA accumulation, and the expression of key enzymes and GLUT1. Our study was the first research confirming that Huaier could impede the anaerobic glycolysis of cancer cells.

Studies have shown that the PI3K/AKT signalling pathway plays an important role in the adaptation of tissue cells to environmental hypoxia, including the induction of glycolysis enhancement and angiogenesis.^43^ During this process, HIF‐1α plays an important mediating role.^44^ A previous study have found that Huaier can play an anti‐tumour effect in gastric cancer cells by acting on the PI3K/AKT signalling pathway.^45^ In line with the previous study, we also observed that the expression of AKT was markedly decreased after administration of Huaier; moreover, the data suggested the decrease of HIF‐1α. Our data implied the inactivation of PI3K/AKT pathway by Huaier. In order to investigate whether Huaier can inhibit the energy metabolism of lung adenocarcinoma cells through the PI3K/AKT/HIF‐1α signalling pathway, we administrated the activator and the inhibitor of the PI3K/AKT pathway. As expected, the expressions of key enzymes involved in glycolysis were all increased by administration of EGF but all decreased by administration of Wortmannin, implying that the activation of PI3K/ATK facilitates the progression of cancer. Interestingly, we observed that silencing of HIF‐1α had no impacts on the expressions of key enzymes regardless of EGF and Wortmannin administration. Thus, we confirmed that Huaier inhibited glycolysis by inhibiting the PI3K/AKT signalling pathway, thereby exerted an anti‐tumour effect. To further confirm the in vitro experiments, we performed the in vivo experiments. A549 cells were injected to the nude mice. After administration with the Huaier granules for 40 days, we were excited that the tumour volume and the weight were both significantly reduced. In addition, the ELISA results demonstrated that the glycolytic enzymes and GLUT1 were all reduced by Huaier granules. The IHC results also confirmed that Huaier indeed reduced the expression of HIF‐1α in tumour tissues. However, the underlying molecular mechanisms still remain unclear. Mechanistically, PI3K/AKT/HIF‐1α signalling pathway has been demonstrated to regulate hypoxia‐induced epithelial‐mesenchymal transition (EMT).^46^ A previous study found that PI3K/AKT/HIF‐1α may promote the progression of human papillomavirus (HPV)‐associated NSCLC by regulating the expression of EMT‐related transcription factors in NSCLC cells.^26^ Hence, further experiments should be conducted to elucidate the molecular mechanism via which Huaier modulates the activity of the PI3K/AKT/HIF‐1α pathway.

More interestingly, FK228, a specific histone deacetylase (HDAC) inhibitor,^47^ has been reported to decrease hypoxia‐induced angiogenesis by suppression of HIF‐1α activity in Lewis lung carcinoma model.^48^ Therefore, we speculated that a combination of Huaier with FK228 might present synergistic effects in lung cancer cells, which may have potential applications in the clinical setting. However, further study should be performed to confirm our speculation.

Taken together, our results suggest that Huaier inhibits the proliferation, migration and energy metabolism of human lung cancer A549 cells through regulating PI3K/AKT/HIF‐1α pathway.

## CONFLICT OF INTEREST

The authors declare that they have no conflicts of interest.

## AUTHOR CONTRIBUTIONS

**XiangLi Liu:** Data curation (lead); Investigation (equal); Supervision (lead); Writing‐review & editing (lead). **Lidan Liu:** Formal analysis (supporting); Project administration (equal); Validation (supporting). **Keyan Chen:** Investigation (equal); Validation (equal). **Lei Sun:** Investigation (equal); Methodology (equal); Software (equal). **Wenya Li:** Investigation (equal); Methodology (equal); Writing‐original draft (equal). **Shuguang Zhang:** Conceptualization (lead); Resources (lead); Writing‐review & editing (lead).

## Data Availability

The datasets used and/or analysed during the current study are available from the corresponding author on reasonable request.
